# Effect of the use of platelet concentrates on new bone formation in alveolar ridge preservation: a systematic review, meta-analysis, and trial sequential analysis

**DOI:** 10.1007/s00784-023-05126-8

**Published:** 2023-07-13

**Authors:** Vito Carlo Alberto Caponio, Laura Baca-González, José González-Serrano, Jesús Torres, Rosa María López-Pintor

**Affiliations:** 1 Department of Clinical and Experimental Medicine, University of Foggia, Foggia, Italy; 2 ORALMED Research Group, Department of Dental Clinical Specialties, School of Dentistry, Complutense University, Madrid, Spain; 3 Department of Dental Clinical Specialties, School of Dentistry, Complutense University, Madrid, Spain; 4 Departamento de Especialidades Clínicas Odontológicas, Facultad de Odontología, Plaza Ramón y Cajal S/N, 28040 Madrid, Spain

**Keywords:** Alveolar ridge preservation, Platelet concentrates, Platelet-rich plasma, Platelet-rich fibrin, Implant treatment, Bone healing, Histomorphometry

## Abstract

**Objectives:**

To investigate the histomorphometric changes occurring in alveolar ridge preservation (ARP) based on the use of different plasma concentrates (PCs) in randomized clinical trials (RCT). There is controversy whether the placement of PCs in ARP is effective in the formation of new bone.

**Materials and methods:**

A systematic review search was conducted in PubMed, Scopus, Web of Science, and Cochrane Database to answer the PICO question: In patients undergoing tooth extraction followed by ARP, do PCs alone in the post-extraction socket in comparison with spontaneous healing improve new vital bone formation percentage in histomorphometric analysis after more than 10 weeks? The risk of bias was assessed and a meta-analysis was conducted.

**Results:**

Of 3809 results, 8 studies were considered suitable for inclusion. A total of 255 teeth were extracted in 250 patients. Regarding the PCs used, ARP was performed with platelet- and leukocyte-rich fibrin (L-PRF) in 120 sockets, and with pure platelet-rich plasma (P-PRP) in 31 sockets and 104 sockets were controlled. PCs improved new bone formation in ARP with respect to the spontaneous healing group (SMD = 1.77, 95%C.I. = 1.47–2.06, *p*-value < 000.1). There were no differences between the different PCs (L-PRF and P-PRP).

**Conclusion:**

The results of this meta-analysis support the efficacy of the use of PCs in new bone formation in ARP. With respect to the different types of PCs studied, no differences were observed.

**Clinical relevance:**

When planning implant surgery after tooth extraction, treatment with PCs should be considered for ARP. Any PC increases new bone formation compared to spontaneous healing.

**Supplementary Information:**

The online version contains supplementary material available at 10.1007/s00784-023-05126-8.

## Introduction

Implantology is a rapid developing specialty of dentistry. Dental implant is the preferred treatment option for patients with missing teeth [1]. Nowadays, there are many studies assessing new techniques to improve treatment protocols, survival, and predictability of implant treatment [2–4].

When a tooth is removed, the alveolar bone undergoes several changes mainly during the first 3 months, that lead to resorption and loss of surrounding bone [5]. Adequate bone quantity and quality is a prerequisite for the success of dental implant treatment [6]. Nowadays, thanks to the studies carried out to improve bone biology, technique, and regenerative materials [7], different treatments have been proposed to reduce bone resorption and improve implant treatment [8–12]. Among these, alveolar ridge preservation (ARP) has shown promising results [13].

ARP includes the use of filling materials in the post-extraction alveolar socket. Many bone substitutes and other biomaterials have been tested for ARP. However, none of them has shown superior results [13–15]. In fact, there are studies highlighting that there is no filling material capable of preventing bone resorption completely [16, 17]. And, in a clinical setting, operators must opt for the one which is able to guarantee the best ARP [18].

In the last decade, autologous platelet concentrates (PCs) have been successfully used to this purpose. PCs are obtained by autologous blood centrifugation [19] and have broad applications in regenerative medicine [20] representing a biocompatible and low-cost option [18]. They were firstly introduced in oral and maxillofacial surgery by Whitman et al. in 1997 [21, 22]. Since then, many protocols have been proposed, resulting in various end products with different characteristics [22, 23], which could influence the amount and kinetics of growth factors release, fibrin architecture, and, therefore, clinical outcomes [24–27]. PC classification is still an important issue in the scientific community [28, 29]. To improve standardization, the classification by Ehrenfest et al. has been introduced [28]. PCs were classified into four groups based on fibrin architecture and leukocyte content. The structure of the fibrin matrix depends on whether or not an anticoagulant is used during preparation. This results in platelet-rich plasma (PRP) if anticoagulant is used and platelet-rich fibrin (PRF) if not. In turn, PRP and PRF may or may not contain leukocytes, giving pure-PRP (P-PRP), leukocyte-rich PRP (L-PRP), pure-PRF (P-PRF), and leukocyte-rich PRF (L-PRF) [24, 30, 31] (Table 1). L-PRF is considered a second generation of PCs [28] and was introduced by Choukroun et al. as a time-saving option compared to PRP [29]. However, PRF may also include leukocytes, which role in inflammation, wound healing, and regeneration is still unclear [26, 32, 33].

**Table 1:** Classification of PCs and relative protocols

	Anticoagulant				Non anticoagulant	
Non-leucocytes	P-PRP				P-PRF	
		*anticoagulant*	*activator*	*centrifugation*		
	PRGF [34]	3.8% sodium citrate	20µL/mL 10% CaCl_2_	580 g x 8 min		
	Nahita [35]	1:9 trisodium citrate, citrate and citrate dextrose acid	0.0025 M CaCl_2_	1500 rpm (280 g) × 7 min		
	ACE [35]	1.5:8.5 trisodium citrate and citrate dextrose acid	0.0025 M CaCl_2_	*2 spins* 1300 rpm (160 g) × 10 min + 2000 rpm (400 g) × 10 min		
Leucocytes	L-PRP				L-PRF	
		*anticoagulant*	*activator*	*centrifugation*		*centrifugation*
	Marx [36]	200µL/mL citrate phosphate dextrose	1:6 10% CaCl_2_ + 10000UI topical bovine thrombin	*2 spins* 5600 rpm x 50 mL/min + 2400 rpm	PRF	3000 rpm x 10 min [37, 38]/ 2700 rpm (408 g) x 12 min [39, 40]
	Curasan [41]	1:8.5 citrate phosphate dextrose and adenosine	Bovine thrombin and calcium chloride [28]	*2 spins* 2400 rpm x 10 min + 3600 rpm x 15 min	A-PRF [39, 42]	1300 rpm (145 g) x 8 min
	Smart PReP [43–45]	EDTA + adenosine-citrate-dextrose	3:1 autogenous thrombin + CaCl_2_	*2 spins* 15 min approx. Automatic two-chamber system	CGF [46–49]	Variable angular speed × 14 min 6 s
					i-PRF	3300 rpm x 2 min [50] 700 rpm x 3 min [51]

PCs have been shown to promote soft tissue healing [52–54], whereas the effects on bone tissue remain controversial [52, 55–58]. While some studies reported improved bone filling, increased bone density, and less ridge width reduction [52, 53, 59–63], others did not [56, 64, 65]. This scenario may be the result of the different protocols used and, therefore, of the different characteristics of each PC. Despite their wide application in clinical practice, there is heterogeneity among different preparation protocols and it is unclear which PC can lead to better results in vital new bone formation.

From this point of view, the aim of this systematic review and meta-analysis was to investigate the histomorphometric changes occurring in ARP based on the use of different PCs in a randomized clinical trial setting.

## Materials and methods

Registration of this systematic review and meta-analysis was performed in the PROSPERO database (Registration No.: CRD42022340941). Preferred Reporting Items for Systematic Reviews and Meta-analyses (PRISMA) guidelines were followed [66].

### Search strategy and database screening

A literature search was conducted in the following databases: PubMed, Scopus, and Web of Science, and Cochrane Database. The first inspection was conducted on June 2, 2022. Retrieved results were updated during last search, performed on December 19, 2022. In each database, a combination of keywords and terms was input to generate an ad-hoc search strategy. The search strategies used for each database are shown in Supplementary Table 1. Resulting references were downloaded and uploaded in EndNote software (EndNote X9.3.2, Clarivate Analytics), which automatically removed the duplicates. Resulting list was furtherly manually screened for extra duplicates.

### Eligibility criteria

The list of references and abstracts resulting from the search were examined. Studies meeting the following inclusion criteria were selected: (1) no restrictions on publication year; (2) English publication language; (3) only randomized clinical trials, also with a split-mouth design; (4) involving patients over 18 years of age; and (5) requiring non-traumatic tooth extraction. Specifically, the eligible study had to address the population (P), intervention (I), comparison (C), and outcome (O) [67] question described below:

- (P): To include patients undergoing tooth extraction followed by ARP.
- (I): ARP was performed by the addition of PCs, for example, PRP or PRF alone in the post-extraction socket.
- (C): Post-extraction sockets were left without any ARP and spontaneous healing was observed.
- (O): Suitable studies evaluated as outcome the effects of healing (ARP with PCs versus spontaneous healing) in terms of new vital bone formation percentage by histomorphometric analysis. The minimum follow-up required of 10 weeks was set to take into account the bone tissue physiology healing process, in which most dimensional alterations take place in the first 3 months following tooth extraction [68, 69], while greater new vital bone formation occurs later [70, 71].

The exclusion criteria were as follows: (1) Studies including only observations taken before 10 weeks of follow-up after the intervention; (2) Studies including third molars post-extraction sockets; (3)Studies realized in patients undergoing head and neck radiotherapy, patients with bone diseases, patients with immune-systemic diseases or uncontrolled diabetes; (4) Studies on cell-line models or animal models; (5) Studies investigating the combination of PCs with other materials or compared to other materials alone and—or without a spontaneous healing group as comparison; and (6) Case reports, case series, cohort, and case-control studies as study designs without a randomization process of patients.

### Reference screening and inclusion

Two authors (VCAC and LBG) independently screened the resulting list for eligible references to be included in this systematic review, according to the inclusion/exclusion criteria listed above. In the first instance, only the title and abstract were assessed, and suitable studies were furtherly evaluated on full-text appraisal. The k-agreement calculation was evaluated to rank the reviewer’s agreement. A k-agreement of 0.77 showed excellent agreement between the two reviewers. A third author (JGS) participated in this phase to resolve discrepancies.

### Data extraction

Independently, two reviewers (VCAC and LBG) performed data extraction based on items collected in ad-hoc extraction Excel sheets. The two reviewers, in a joint meeting with a third reviewer (JGS), merged the extraction Excel files to find for discrepancies, which were fixed in the same meeting after full-text evaluation.

The following information were recorded:

- First author, year of publication, and country where the study was performed.
- Study design.
- Type of PCs: P-PRP, P-PRF, L-PRP, or L-PRF.
- Characteristics of the patients: included number of patients, gender, mean age (Standard Deviation (S.D.) or range), smoking habit, and periodontal status.
- Information about the tooth extraction: teeth extracted, the reason for extraction, information about the extraction procedure (with or without flap, type of suture), and the number of walls in the socket.
- Information about the biopsy sampling, histomorphometric protocol, outcomes collected, and follow-up(s) in weeks.
- Platelet concentrates protocols: use of anticoagulants, use of activators, and cycles of centrifugation, speed, and time.
- New bone formation: number of tooth sockets treated for each group, new bone formation percentage expressed as mean and S.D.

### Risk of bias assessment

The analysis of the risk of bias of the studies included was performed according to the Cochrane Risk of Bias in randomized interventional studies tool (RoB 2) in the last version, dated 22 August 2019 [72]. The assessment was specific to estimate the relative effect of two interventions on a target outcome. All participants underwent atraumatic tooth extraction and ARP using PCs (intervention) versus the physiological healing by a regular blood clot (control) in order to assess the percentage of new formed bone (outcome).

Concerning split-mouth design studies, RoB assessment was performed adopting an extension of the CONSORT guidelines for withing person trials [73].

RoB was performed independently by two authors (VCAC and LBG) and disagreements were solved in a joint meeting with a third reviewer (JGS).

### Statistical analysis and data pooling

A meta-analysis was performed for pooled percentages of new vital bone formation for both RCTs and split-mouth RCT design studies. A meta-epidemiological study did not provide sufficient evidence for a difference in intervention effect estimates between parallel-arm RCTs and RCT-split mouth design studies, so a meta-analysis was performed including both study designs. However, subgroup analysis was also performed [74]. Data were input as mean values of percentages of new vital bone formation with respective S.Ds. and sample size for the control group versus the test group. In the study of Castro et al. [39] two different protocols of PCs were used, however, resulting in both in L-PRF. For this reason, the means and S.Ds. of both groups were combined in contrast to the control, employing the formula from the Cochrane Handbook for Systematic Reviews of Interventions version 6.3 [75].

Overall standardized mean difference (SMD) and relative 95% confidence interval (95% C.I.) were estimated by Hedges’ g weighted data and were graphically represented by forest plots in a fixed or random effect model, based on heterogeneity. Heterogeneity between studies was assessed by Cochran’s Q test and quantified by the I^2^ index. For I^2^ values higher than 50%, a random model was set, whether for lower values a fixed effect model was adopted [76]. Heterogeneity was furtherly evaluated by investigating differences among studies and was grouped as moderators, in particular, sensitivity analysis was run for (1) follow-up(s) in weeks; (2) type of PCs as L-PRF and P-PRP; (3) publication year; and (4) study design as RCT versus RCT split-mouth. ANOVA Q-test was used to assess statistically significant differences among subgroups [77].

To inspect the influence of individual studies on overall standardized mean difference, leaving one out method was employed [78]. In the last instance, a funnel plot was generated to graphically visualize the publication bias and was integrated by trim and fill analysis [79], Egger’s test [80], and the safe N test [81].

Trial Sequential Analysis [82] was employed to evaluate the strength of evidence and adjust for potential errors. The TSA software was used in its version 0.9 beta from the Copenhagen Trial Unit. The analysis set specific values for type 1 and 2 errors (5% and 10%) and used these values to calculate trial sequential monitoring boundaries, futility boundaries, and the required information size (RIS) [83, 84]. The mean difference to generate RIS was user-defined with the objective of detecting a mean difference of 7% of new vital bone formation between the test and control. The variance was based on an empirical model. The study also applied a model variance-based approach to correct for heterogeneity and used a graphical evaluation to determine if the cumulative Z-curve met defined thresholds [85].

## Results

### Search strategy and screening

**Fig. 1:**
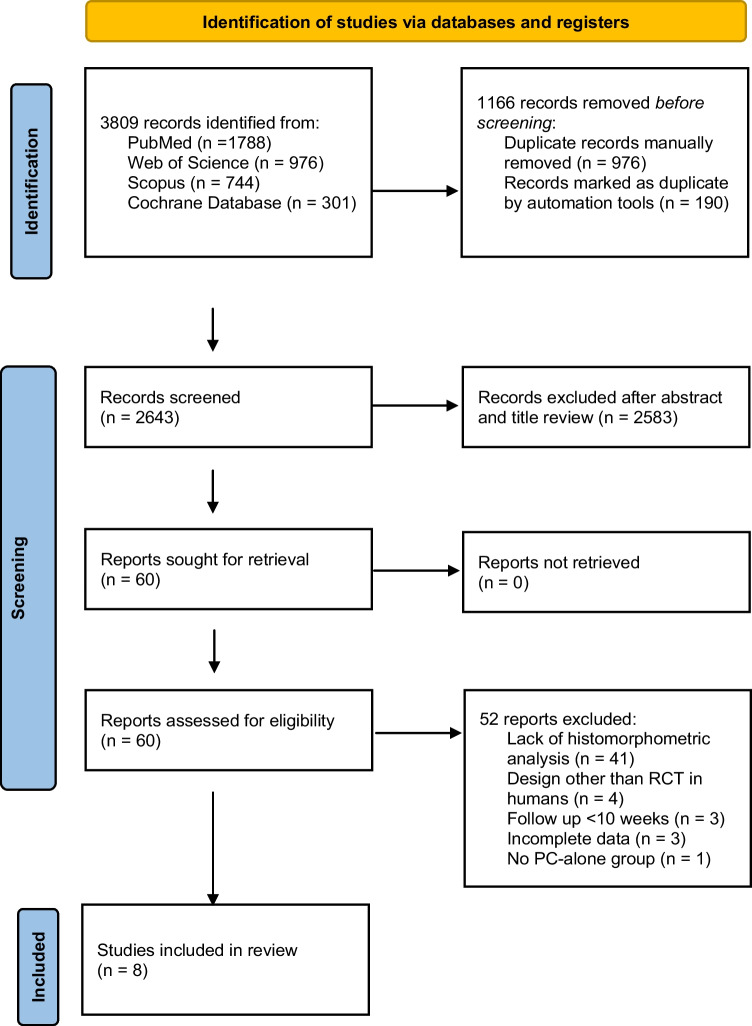
PRISMA flowchart with detailed selection process

The last search in the mentioned databases yielded 3809 results (PubMed = 1788, Scopus = 744, Web of Science = 976, Cochrane Database = 301). These references were integrated into the EndNote reference software tool (Endnote X9.3.2, Clarivate Analytics). Once duplicates were removed, the titles and abstracts of a total of 2376 references were examined and 2316 were excluded. Sixty references were evaluated in the full text, and of these 52 studies were excluded (the list and rationale for exclusion are summarized in Supplementary Table 2). In the end, 8 studies were considered suitable for inclusion in the systematic review and meta-analysis. Figure 1 shows the flowchart.

### General characteristics of included studies

The selection process resulted in 8 studies eligible for meta-analysis [37, 39, 40, 42, 57, 86–88]. The studies were published between 2015 and 2021. The studies were mostly performed in Europe [39, 42, 57, 86, 87], but two studies were performed in Brazil [40, 88], and one in South Africa [37].

Clinical characteristics are summarized in Table 2. A total of 6 studies adopted a RCT design, and two studies a split-mouth design [37, 39]. ARP was most frequently performed in the sockets of single-rooted teeth in the upper anterior maxilla. L-PRF was the most commonly investigated PC and only two studies employed PRP [57, 87]. All studies used sutures to stabilize clots, most without primary closure intention. Two studies completely covered the extraction site with a mucoperiosteal flap [42, 86]. One study sealed the sockets with L-PRF membranes placed in full-thickness buccal and lingual/palatal envelopes [39]. Only one study included heavy smokers (≥ 10 cigarettes/day), representing a total of 6 patients evenly distributed across the control and test groups [57]. This was the only study [57] that specifically included patients with periodontal disease, present in 52.7% and 45.8% of the patients assigned to the experimental and control groups, respectively. The percentage of new bone formation was evaluated after 10–24 weeks of follow-up.

**Table 2:** General characteristics of the studies and patients included in these studies and of the extractions performed

Author, year, country	Study desing	Type of PCs	Patients (n) and sex		Mean age (SD or range)		Teeth extracted	Reason for extraction	Extraction procedure	Number of walls in the socket	Smoking habit included	Periodontal status
			Test	Control	Test	Control						
Anitua et al., 2015 Spain	RCT	P-PRP	30 (19 M, 17F)	22 (10 M, 14F)	57 (29–74)	53 (18–67)	Mandibular molars	Endodontic treatment failure, severe bone loss, non-restorable tooth, periapical abscess, occlusal interferences	Flapless (except 1 case in P-PRP group)	Sockets without bone defects	Yes	Patients with periodontal disease: 52.7% (control) and 45.8% (test)
Ivanova et al., 2019 Bulgaria	RCT	L-PRF	23	12	NA	NA	NA	NA	Mucoperiosteal flap	NA	< 10 cigarettes/day	Good oral hygiene
Canellas et al., 2020 Brazil	RCT	L-PRF	24 (9 M, 15F)	24 (12 M, 12F)	43.6 (18–69)	46.1 (26–68)	Non-molars	Endodontic treatment failure, untreated caries, root fracture, unfavorable prosthetic support	Flapless	Sockets without bone defects	No	Good oral hygiene
Stumbras et al., 2020 Lithuania	RCT	P-PRP	10 (4 M, 6F)	10 (3 M, 7F)	48 (13)	51 (14)	Premaxilla	Endodontic failure, fracture, periodontal problem, caries	Flapless	> 50% of the buccal bone height	< 10 cigarettes/day	Active periodontitis excluded
Ivanova et al., 2021 Bulgaria	RCT	L-PRF	30	30	42.93 (10.89)	40.80 (10.50)	NA	Crown and root fracture or destruction, root resorption, periodontitis	Mucoperiosteal flap	Sockets without bone defects	< 10 cigarettes/day	Good oral hygiene
Martins et al., 2021 Brazil	RCT	L-PRF	5	5	NA	NA	Premaxilla	NA	Intrasulcular incision	Sockets without bone defects	No	Good oral hygiene
Du Toit et al., 2016 South Africa	RCT split mouth	L-PRF	4 (3 M, 1F)		39.5 (5.67)		Premaxilla	Hopeless teeth	Flapless	Plates preserved during extraction	No	Good oral hygiene Active periodontitis excluded
Castro et al., 2021 Belgium	RCT split mouth	L-PRF	21 (6 M, 15F)		64.4 (12)		Premaxilla	NA	Full thickness envelope to stabilize L-PRF membrane	Buccal bone plate preserved	< 10 cigarettes/day	NA

NA, Not available

Biopsy sampling was performed at the time of implant placement in all the studies. Before implant placement, a trephine bur was employed to collect the bone sample. The diameter ranged from 2 to 3 mm and the length from 4 to 7 mm, but Ivanova et al. studies and Castro et al. did not report biopsy sample length. Histomorphometric protocol differed among studies. The detailed histomorphometric protocol is reported in Table 3.

**Table 3:** Biopsy characteristics and protocol to perform histomorphometry

Author, year, country	PRP or PRF	Biopsy sampling	Histomorphometric protocol	Follow up (weeks)
Anitua et al., 2015 Spain	P-PRP	At implant placement – 2.25 mm trephine bur – 5 mm length	Fixation in formalin without decalcification Inclusion in methacrylate resin Staining with hematoxylin–eosin and May–Grünwald–Giemsa	10–12 w
Ivanova et al., 2019 Bulgaria	L-PRF	At implant placement – 2.5 mm trephine bur – NS length	Fixation in 10% formalin. Dehydration with xylene 3–4 microns sections were incorporated in paraffin blocks Staining with hematoxylin–eosin	16 w
Canellas et al., 2020 Brazil	L-PRF	At implant placement – 2 mm trephine bur – 6–8 mm length	Decalcification and staining with Goldner's trichrome and hematoxylin–eosin	12 w
Stumbras et al., 2020 Lithuania	P-PRP	At implant placement – 2.5–3 mm trephine bur – 4–5 mm length	Fixation in 4% formalin. Dehydration in ascending concentration of ethanol. Un-decalcified inclusion in methyl methacrylate resin Staining with hematoxylin–eosin and May Grünwald-Giemsa	12 w
Ivanova et al., 2021 Bulgaria	L-PRF	At implant placement – 3 mm trephine bur – NS length	Fixation in 10% formalin Decalcification with EDTA Dehydration in ascending concentration of alcohol (70% ethyl alcohol, 95% ethyl alcohol, 99% ethyl alcohol and clarified with xylene) 3–4 microns sections were incorporated in paraffin blocks Staining with hematoxylin–eosin	16 w
Martins et al., 2021 Brazil	L-PRF	At implant placement – 2 mm trephine bur – 7 mm length	Fixation in 4% paraformaldehyde Decalcification with 10% EDTA 7 microns sections were incorporated in paraffin blocks Staining with hematoxylin–eosin	24 w
Du Toit et al., 2016 South Africa	L-PRF	At implant placement – 2.8 mm trephine bur – 7 mm length	Fixation in 70% ethanol without decalcification Dehydration 10 microns sections were embedded in Technovit 7200 media resin blocks Staining with methylene blue-basic fuchsin	12 w
Castro et al., 2021 Belgium	L-PRF	At implant placement – 2 mm trephine bur – NS length	Fresh frozen in liquid nitrogen and kept at -80 °C Fixation in 4% paraformaldehyde. Decalcification with 0.5 M EDTA/PBS. Dehydration. 4 microns sections were embedded in paraffin blocks. Staining with hematoxylin–eosin	12 w

Concerning PCs preparation (Table 4), different centrifugation protocols emerged, both in time of centrifugation and revolutions per minute of the rotor (rpm) or relative centrifugal force (g). Even in similar reported rpm, g changed because of the different radius of rotors employed for the centrifugation.

**Table 4:** Platelet concentrates protocols in the different studies included

Author, year, country	Platelet Concentrate	Group	Anticoagulant	Activator	Cycles of Centrifugation, speed (rpm or g) and time (min)
Anitua et al., 2015 Spain	PRGF	P-PRP	3.8% sodium citrate	400 μL of 10% CaCl_2_	1 580 g 8 min
Du Toit et al., 2016, South Africa	PRF	L-PRF	NA	NA	1 3000 rpm 10 min
Ivanova et al., 2019 Bulgaria	A-PRF	L-PRF	NA	NA	1 1300 rpm 12 min
Canellas et al., 2020 Brazil	L-PRF	L-PRF	NA	NA	1 2700 rpm 12 min
Stumbras et al., 2020 Lithuania	PRGF	P-PRP	3.8% sodium citate	20 uL of 10% CaCl_2_ per mL of plasma	NS Manufacturer instructions
Castro et al., 2021, Belgium	L-PRF	L-PRF	NA	NA	1 2700 rpm (408 g) 12 min
Castro et al., 2021, Belgium	A-PRF	L-PRF	NA	NA	1 1300 rpm (145 g) 8 min
Ivanova et al., 2021 Bulgaria	A-PRF	L-PRF	NA	NA	1 1300 rpm (200 g) 8 min
Martins et al., 2021 Brazil	PRF	L-PRF	NA	NA	1 400 g 12 min

*NA*, Not available

In total 255 teeth were extracted and ARP was performed with L-PRF in 120 sockets and with P-PRP in 31 sockets. On the other hand, 104 sockets were left healing spontaneously. These procedures were performed in 225 patients from studies with an RCT design and 25 patients from studies with a split-mouth RCT design. Previous information and the mean and S.Ds of new bone formation percentages of each group in all included studies are reported in Table 5.

**Table 5:** New bone formation reported in the included studies, differences for PRP/PRF group versus control and number of treated sockets

Author, year, country	PRP or PRF	Number of sockets		New bone formation % (S.D.)	
		Test	Control	Test	Control
Anitua et al., 2015 Spain	P-PRP	21	5	63.10 (13.8)	35.60 (35.3)
Ivanova et al., 2019 Belgium	L-PRF	19	8	60.48 (9.88)	36.93 (14.94)
Canellas et al., 2020 Brazil	L-PRF	22	22	55.96 (11.97)	39.69 (11.13)
Stumbras et al. 2020 Lithuania	P-PRP	10	10	75.50 (16.3)	46.50 (15.2)
Ivanova et al. 2021 Bulgaria	L-PRF	30	30	60.79 (9.72)	39.04 (10.89)
Martins et al. 2021 Brazil	L-PRF	5	5	54.20 (4.31)	40.60 (5.98)
Du Toit et al., 2016 South Africa	L-PRF	4	4	50.70 (13.3)	47.90 (18.1)
Castro et al., 2021 Belgium	L-PRF	20	20	47.70 (7.9)	34.70 (6.9)
Castro et al., 2021 Belgium	L-PRF	20	20	54.50 (5.6)	34.70 (6.9)

### Risk of bias

Table 6 summarizes the risk of bias results for each study. Among RCTs, 3 studies showed some concerns in the randomization process. No information in this regard was reported in the Ivanova et al. study [86]. While in the other two studies, it was unclear whether the allocation sequence was concealed until participants were enrolled and assigned to interventions [87, 88]. When assessing “deviation from intervention”, no indications were provided in the study of Ivanova et al. [86], for this reason, this study was evaluated at high risk of bias. All the studies resulted in a low risk of bias in the “missing outcome data” item. The study by Ivanova et al. was the only one with a high risk of bias in the item “measurement of the outcome”, as it did not indicate whether the assessors collecting the results were aware of the intervention received by the study participants [86]. The “Selection of reported results” item had some concerns in three studies, since a pre-specified analysis plan for data was not indicated [42, 86, 88]. Overall, only one study resulted in a low risk of bias [40], three reported some concerns [42, 57, 87], while two studies were at high risk of bias [86, 88].

**Table 6:** Risk of bias assessment according to RoB 2, showing evaluation per item and overall ranking

Study	Randomization process	Deviation from intervention	Missing outcome data	Measurement of the outcome	Selection of reported result	Overall rating
Anitua et al., 2015 Spain [57]	Low	Some concerns	Low	Low	Low	Some concerns
Ivanova et al., 2019 Bulgaria [86]	Some concerns	High	Low	High	Some concerns	High
Canellas et al., 2020 Brazil [40]	Low	Low	Low	Low	Low	Low
Stumbras et al., 2020 Lithuania [87]	Some concerns	Low	Low	Low	Low	Some concerns
Ivanova et al., 2021 Bulgaria [42]	Low	Low	Low	Low	Some concerns	Some concerns
Martins et al., 2021 Brazil [88]	Some concerns	Low	Low	Some concerns	Some concerns	High

For Castro et al. [39] and Du Toit et al. [37] 46 items were evaluated from merging the standard CONSORT guideline checklist and the one for withing person trial [73]. Du Toit et al. and Castro et al. scored respectively 38 and 36 points. Du Toit et al. failed to report “other information” because the description of registration, protocol, and funding was missing. Castro et al. failed to provide information in different subfields of “Methods” (items and scores are collected in Supplementary Tables 3 and 4 for each study). Both studies were attributed to “some concerns” [37, 39].

### Meta-analysis and trial sequential analysis

**Fig. 2:**
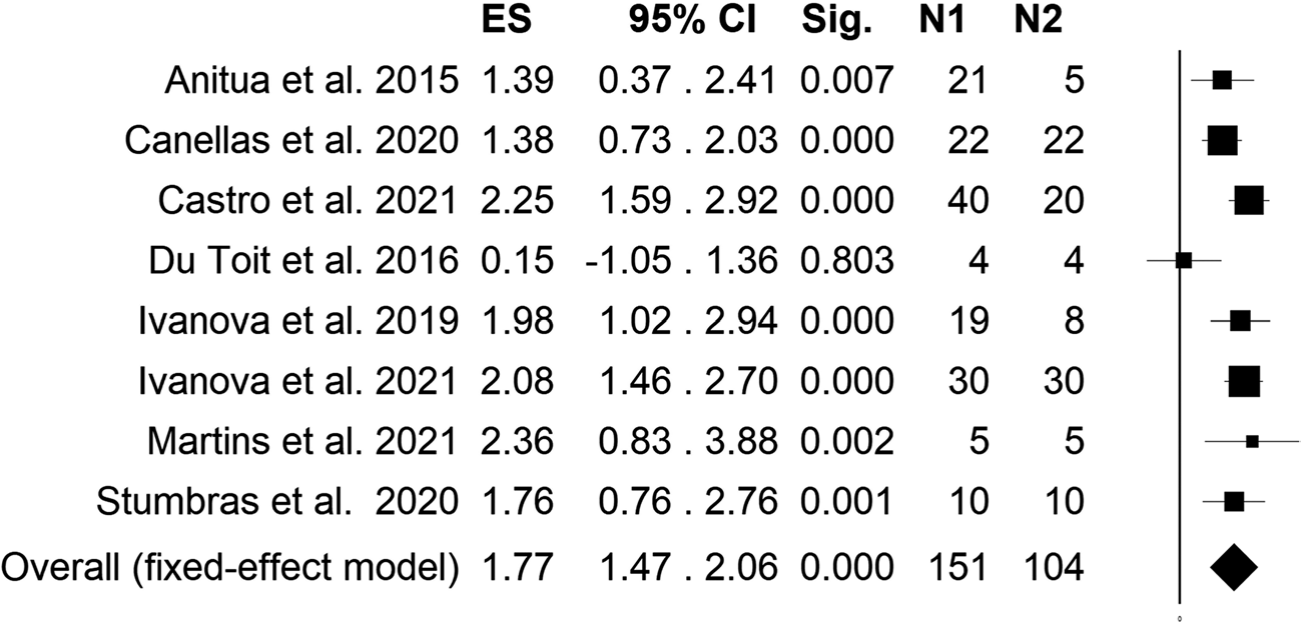
Forest plot depicting overall standardized mean difference estimation for meta-analysis comparing PCs group (N1) vs spontaneous healing (N2), fixed effects model *I*^2^ = 44.05

**Fig. 3:**
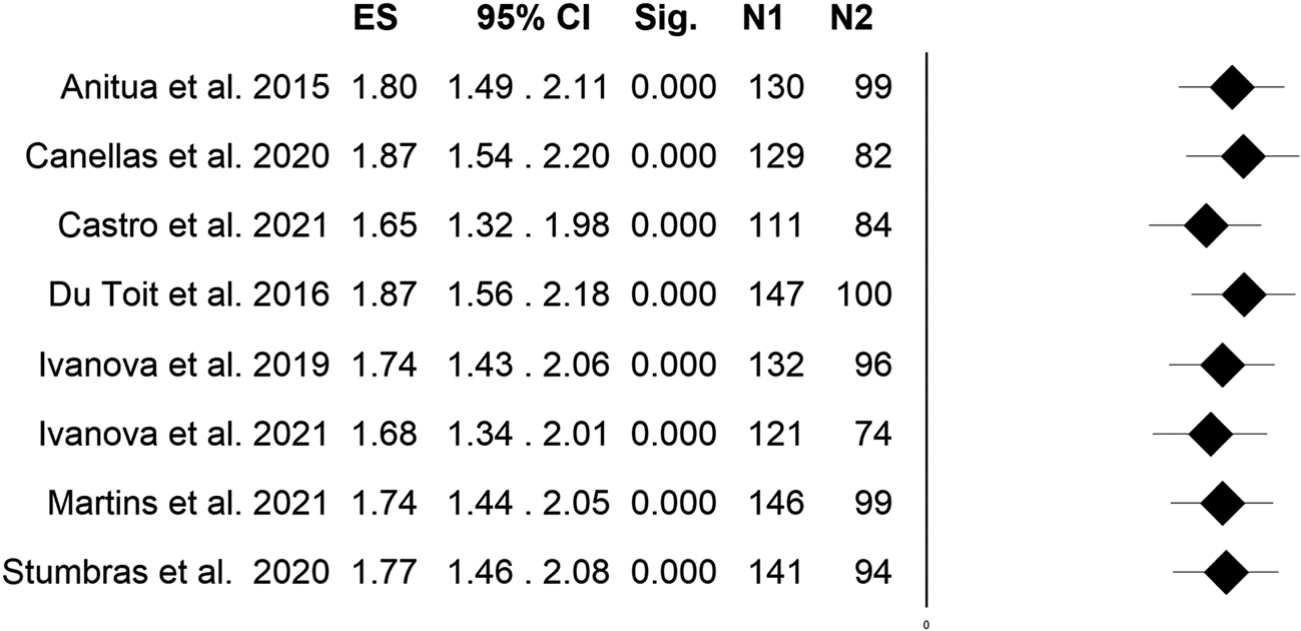
Forest plot depicting overall standardized mean difference estimation for meta-analysis comparing PCs group (N1) vs spontaneous healing (N2). Standardized mean differences are shown after leave-one out method

Heterogeneity results showed average heterogeneity among studies (I^2^ = 44.05%). Fixed effect model meta-analysis showed a positive SMD in patients undergoing ARP with PCs with respect to the spontaneous healing group (SMD = 1.77, 95% C.I. = 1.47–2.06, *p*-value < 000.1, Fig. 2). Heterogeneity among studies was furtherly investigated by leave-one-out method (Fig. 3), which showed different SMD after elimination of Castro et al. (SMD = 1.65, 95% C.I. = 1.32–1.98, *p*-value < 0.001) and Du Toit et al. (SMD = 1.87, 95% C.I. = 1.56–2.18, *p*-value < 000.1) studies, which were the only two studies with a split-mouth design.

Subgroup meta-analysis considering RCTs and split-mouth design RCTs separately showed that study design accounted partially for heterogeneity, since I^2^ statistics resulted in 0 only in the RCT subgroup. Similar overall fixed SMD was achieved from RCTs (SMD = 1.77, 95% C.I. = 1.42–2.11, *p*-value < 0.001; vs split-mouth SMD = 1.76, 95% C.I. = 1.18–2.35, *p*-value < 0.001; ANOVA Q-test *p*-value = 0.987—Supplemental Fig. 1). Indeed, differences emerged when investigating PC subtypes, in particular L-PRF and P-PRP. The absence of heterogeneity was found in the P-PRP subgroup analysis. However, the L-PRF subgroup included both split-mouth designs, raising I^2^ = 58.10%. However the difference in SMD between P-PRP and L-PRF was not statistically significant (L-PRF SMD = 1.75, 95% C.I. = 1.21–2.28, *p*-value < 0.001; vs P-PRP SMD = 1.58, 95% C.I. = 0.87–2.29, *p*-value < 0.001; ANOVA Q-test *p*-value = 0.71—Supplemental Fig. 2).

**Fig. 4:**
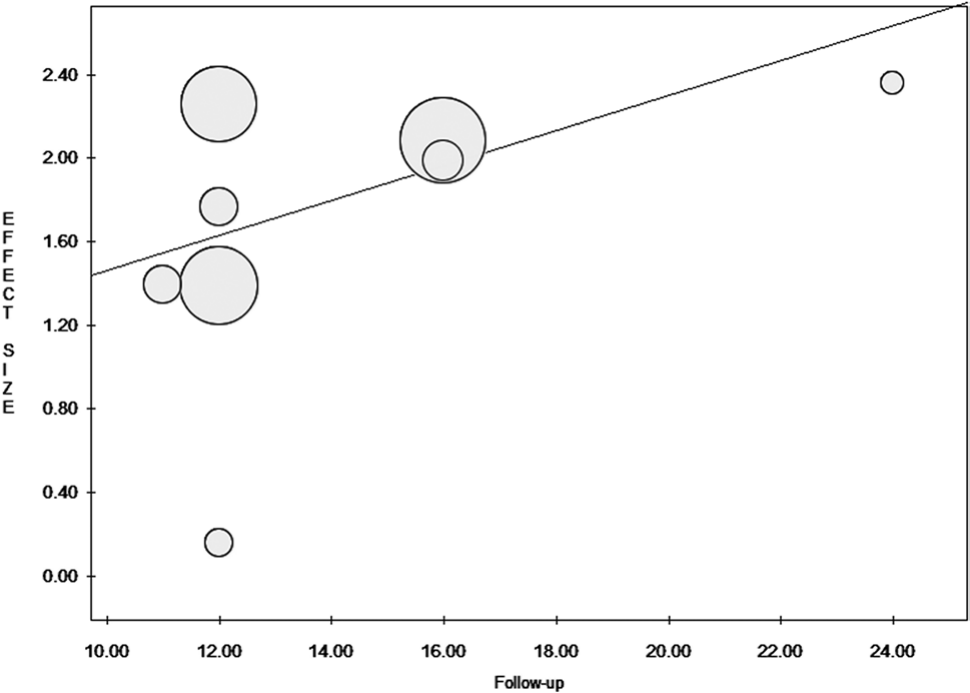
Meta-regression correlating ESs and follow-up. The study of Martins et al. [88] was the only study with a follow-up of 24 months and reported highest ES, 2.36. Du Toit et al. [37] reported lowest ES, 0.15

Surprisingly, meta-regression for publication year found a statistically significant difference in SMDs (fixed effect model *p*-value = 0.048). Meta-regression did not find a statistically significant difference between SMDs and follow-up, besides it seemed to increase for studies with higher follow-ups (fixed effect model *p*-value = 0.27—Fig. 4).

**Fig. 5:**
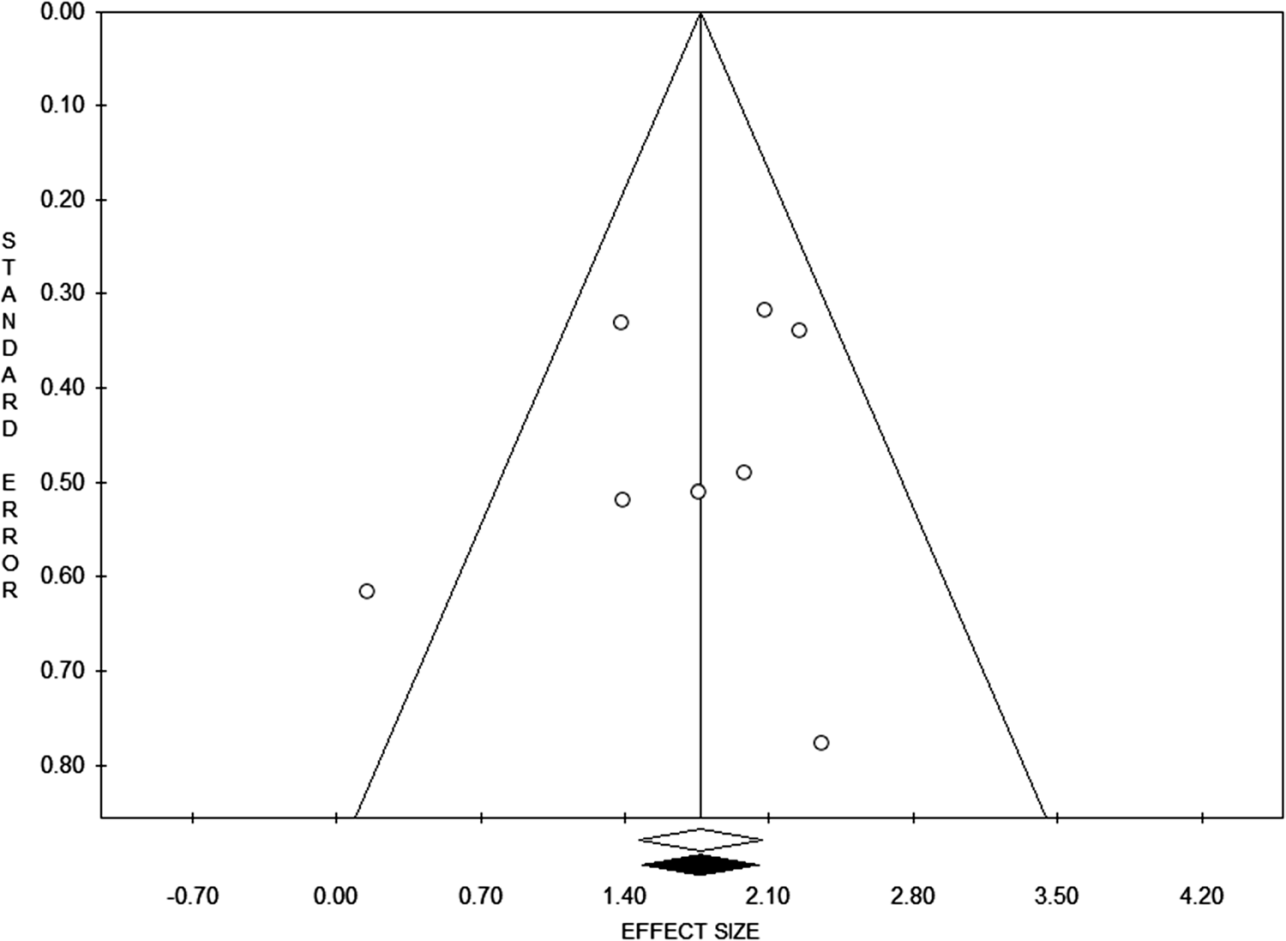
Funnel plot showing each study standardized mean difference related standard error

The absence of publication bias was highlighted by the trim and fill method which found 0 trimmed studies and graphically represented by funnel plot (Fig. 5). Also Egger’s linear regression test showed an absence of publication bias (*p*-value = 0.44). Moreover, the safe N test required the publication of 239 studies to revert the current overall SMD *p*-value.

**Fig. 6:**
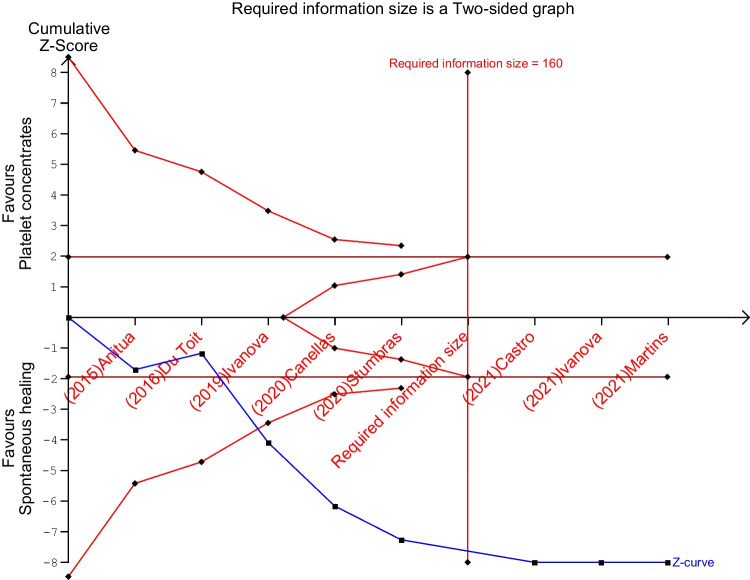
Trial sequential analysis. Blue line represents cumulative z-curve after the addition of each study in chronological order. Red perpendicular line represents the required information size

TSA analysis approved results from meta-analysis after the addition of the third study and confirmed the efficacy of PCs versus spontaneous healing in determining a greater formation of vital bone percentage, since the cumulative z-curve crossed the monitoring boundaries. After the addition of the sixth study, RIS was reached, providing a conclusive statistically significant difference in the results for this meta-analysis (Fig. 6).

## Discussion

This systematic review and meta-analysis with TSA showed conclusive results in the efficacy of PCs in new bone formation in ARP with respect to the spontaneous healing group (SMD = 1.77, 95% C.I. = 1.47–2.06, *p*-value < 000.1). Furthermore, the results of our study observed that there was no difference between the use of the different PCs included (P-PRP and L-PRF).

After tooth extraction, significant alveolar bone remodeling has been documented, leading to a decrease in alveolar height and width mainly at the expense of the vestibular plate [5]. This situation could influence the proper three-dimensional placement of implant-supported restorations as well as the esthetics mainly in the anterior sector. Therefore, one of the main goals of oral implantology is the preservation of the remining healthy bone after tooth extraction using highly predictable procedures [6]. After the first RCT about the success of P-PRP in APR compared to spontaneous healing by Anitua et al. in 2015 [57], new published evidence support the use of PCs in ARP. In the network meta-analysis published by Canellas et al. more than twenty materials were compared in ARP. L-PRF showed no statistically significant differences in ARP with the other best-performing graft materials (MP3®, Apatos®, Gen-Os® and Bond-apatite®).

The inconclusive results about the use of PCs in ARP may be due to the low number of studies using PCs without a xenograft, since most of the studies included combinations of materials which could modify the biological properties of PCs. According to previous studies, the use of PCs have certain advantages such as rapid reabsorption and formation of new trabecular bone while promoting healing due to abundant growth factors. Possibly, a good choice is the combination of a low resorbable material, as xenografts, that preserves the volume of the socket, together with another material that favors the formation of new bone to promote osseointegration and primary stability [89]. This approach is supported by other studies who have reported that ARP with any material is superior to spontaneous healing, and the use of different scaffold materials could favor the reduction of postextraction socket volume [13]. In addition, the application of PCs could improve the healing of the area increasing the formation of new bone [13, 18, 89]. Our study shows that the use of PCs in ARP, regardless of the type of PCs used, improves bone formation compared to spontaneous healing. This amount of neoformed bone must be taken into account in terms of its therapeutic significance. In any case, it should be noted that in our meta-analysis alveolar remodeling measures were not taken into consideration. This could be a limitation of this study, as current knowledge in ARP considers the formation of new vital bone and the preservation of ridge dimension together. This is because both processes can influence primary and secondary implant stability and osseointegration. To overcome this limitation, it is necessary to include studies that combine a xenograft with PCs that may increase the formation of new vital bone, compared to allograft alone. But, this does not allow us to know what effect PCs alone have on bone regeneration [90, 91].

To evaluate differences between the different PCs, it is necessary to unravel their biological behavior. Bone regeneration needs a complex coordination between cytokines, proteins, and grow factors (GFs), and the controlled release of these bioactive substances seems to play a major role in this process. Many studies analyze the release kinetics of GFs from PCs, but there is enormous variability among authors in reporting these results. It has been suggested that these observed differences in the controlled release of GFs from different PCs depend on the architecture of the fibrin matrix and its degree of cross-linking. Some studies consider that L-PRF produces a progressive release of growth factors, whereas PRP triggers a cascade release in the first hours [25, 31]. In contrast, other studies suggest the opposite based on a more rapid degradation of the fibrin matrix of L-PRF due to proinflammatory metalloproteinases produced by leukocytes [26]. In this meta-analysis, further considerations emerged. The scientific scenario offers a wide number of PC types and protocols, increasing heterogeneity. Changes in rotor diameter, number of spins, time and speed of centrifugation could contribute to different biologic characteristics of PCs, even though classified in the same group as P- L-PRP/PRF. In a rat model, different protocols for L-PRP preparation were employed, leading to differences in platelets and minerals concentrations, which impacted significally in reducing the bone defects [27]. This phenomenon, however, is still controversial and limited to short follow-up of bio-molecular events [26] since bone healing is a longer process [68–71]. Indeed, meta-regression showed an increased of new bone formation when measurements were done at longer follow-ups. In any case, no differences could be found in this study between the two PCs analyzed (P-PRP and L-PRF). Therefore, the PC with the simplest and cheapest technique should be used. Normally, the PRF technique is simpler but it has the disadvantage that it is not useful to vehicle other biomaterials. Therefore, depending on the ARP technique to be performed, the clinician will have to decide which one to use.

This study has certain limitations. Only two studies analyzed the use of P-PRP, so that in the future it would be convenient to perform more studies with this type of PC. Another limitation is that PCs protocols differed among studies and the outcome was observed at different follow-ups. Also the inclusion of two split-mouth RCTs can have an impact in the results. But, different analyses have been performed to minimize these issues. It is also worth considering that only new vital bone formation analyzed by histomorphometry was evaluated as an outcome in this study. Although it is expected that a higher percentage of vital bone will result in more bone tissue being available at the time of implant surgery, this is uncertain. And there were no other variables associated with clinical, function or treatment success analyzed in all the studies [92, 93]. It is worth considering that only Anitua et al. collected patient-reported outcomes among the included studies. The P-PRP group showed a statistically significant reduction in reported pain in the first week after extraction, supported by a lower inflammation score. It is necessary to analyze in future studies clinical variables such as changes in height and width, as well as outcomes associated with the patient's perspective such as pain or oral health-related quality of life.

Another limitation of this study is the great heterogeneity, since certain characteristics were different among the studies. These included data on smoking, periodontal status, included teeth (uni- or multiradicular), number of bony walls of the defects, and type of surgery (with or without flap). All these differences constitute potential confounding factors.

## Conclusion

In conclusion, PCs are widely used in clinical practice, despite poor standardization and deep knowledge of molecular events happening in the healing process. Current evidence coming from this meta-analysis of RCTs supports the efficacy of PCs in the new bone formation process, compared to spontaneous healing. P-PRP has been tested in only two RCTs, while L-PRF has found wide application. However, subgroup analysis did not show a statistical difference between these two different PCs.

## Supplementary Information

Below is the link to the electronic supplementary material.

Supplementary file1 (DOCX 133 KB)

## Data Availability

Data to perform these studies are available after a justified request to the authors.
