# Supplementary material for: Effect of the use of platelet concentrates on new bone formation in alveolar ridge preservation: a systematic review, meta-analysis, and trial sequential analysis
Source: Clin Oral Investig. 2023 Jul 13;27(8):4131–46. doi: 10.1007/s00784-023-05126-8 (PMC10415431; doi:10.1007/s00784-023-05126-8)

**Supplemental Table 1:** Search strategy, specific for each screened database

| **Search strategy** |
| --- |
|  |
| **PubMed** |
| ((Platelet concentrates) OR (platelet-rich fibrin) OR (PRF) OR (platelet-rich plasma) OR (PRP) OR (leukocyte platelet-rich fibrin) OR (L-PRF) OR (leukocyte platelet-rich plasma) OR (L-PRP) OR (pure platelet-rich fibrin) OR (P-PRF) OR (pure platelet-rich plasma) OR (P-PRP) OR (plasma-rich in growth factors) OR (PRGF) OR (injectable platelet-rich fibrin) OR (I-PRF) OR (growth factors) OR (platelet-derived growth factors)) AND ((Socket preservation) OR (Alveolar ridge preservation) OR (Post-extraction preservation) OR (Socket healing) OR (Preserved socket) OR (Extraction socket) OR (Alveolar bone preservation) OR (Alveolar bone sockets) OR (Ridge preservation) OR (Extraction socket preservation) OR (Alveolar ridge socket preservation)) |
|  |
| **Scopus** |
| TITLE-ABS-KEY ( ( ( platelet  AND concentrates )  OR  ( platelet-rich  AND fibrin )  OR  ( prf )  OR  ( platelet-rich  AND plasma )  OR  ( prp )  OR  ( leukocyte  AND platelet-rich  AND fibrin )  OR  ( l-prf )  OR  ( leukocyte  AND platelet-rich  AND plasma )  OR  ( l-prp )  OR  ( pure  AND platelet-rich  AND fibrin )  OR  ( p-prf )  OR  ( pure  AND platelet-rich  AND plasma )  OR  ( p-prp )  OR  ( plasma-rich  AND in  AND growth  AND factors )  OR  ( prgf )  OR  ( injectable  AND platelet-rich  AND fibrin )  OR  ( i-prf )  OR  ( growth  AND factors )  OR  ( platelet-derived  AND growth  AND factors ) )  AND  ( ( socket  AND preservation )  OR  ( alveolar  AND ridge  AND preservation )  OR  ( post-extraction  AND preservation )  OR  ( socket  AND healing )  OR  ( preserved  AND socket )  OR  ( extraction  AND socket )  OR  ( alveolar  AND bone  AND preservation )  OR  ( alveolar  AND bone  AND sockets )  OR  ( ridge  AND preservation )  OR  ( extraction  AND socket  AND preservation )  OR  ( alveolar  AND ridge  AND socket  AND preservation ) ) ) |
|  |
| **Web of Science** |
| ((Platelet concentrates) OR (platelet-rich fibrin) OR (PRF) OR (platelet-rich plasma) OR (PRP) OR (leukocyte platelet-rich fibrin) OR (L-PRF) OR (leukocyte platelet-rich plasma) OR (L-PRP) OR (pure platelet-rich fibrin) OR (P-PRF) OR (pure platelet-rich plasma) OR (P-PRP) OR (plasma-rich in growth factors) OR (PRGF) OR (injectable platelet-rich fibrin) OR (I-PRF) OR (growth factors) OR (platelet-derived growth factors)) AND ((Socket preservation) OR (Alveolar ridge preservation) OR (Post-extraction preservation) OR (Socket healing) OR (Preserved socket) OR (Extraction socket) OR (Alveolar bone preservation) OR (Alveolar bone sockets) OR (Ridge preservation) OR (Extraction socket preservation) OR (Alveolar ridge socket preservation)) |
|  |
| **Cochrane database** |
| ((Platelet concentrates) OR (platelet-rich fibrin) OR (PRF) OR (platelet-rich plasma) OR (PRP) OR (leukocyte platelet-rich fibrin) OR (L-PRF) OR (leukocyte platelet-rich plasma) OR (L-PRP) OR (pure platelet-rich fibrin) OR (P-PRF) OR (pure platelet-rich plasma) OR (P-PRP) OR (plasma-rich in growth factors) OR (PRGF) OR (injectable platelet-rich fibrin) OR (I-PRF) OR (growth factors) OR (platelet-derived growth factors)) AND ((Socket preservation) OR (Alveolar ridge preservation) OR (Post-extraction preservation) OR (Socket healing) OR (Preserved socket) OR (Extraction socket) OR (Alveolar bone preservation) OR (Alveolar bone sockets) OR (Ridge preservation) OR (Extraction socket preservation) OR (Alveolar ridge socket preservation)) in Title Abstract Keyword |

**Supplemental Table 2:** References excluded and reason for exclusion.

| **Author, year, country** | **PRP or PRF** | **Not included and reason for exclusion** |
| --- | --- | --- |
| Anitua, 1999 | PRGF | Not included: PRGF is mixed with bone |
| Sammartino et al., 2005 | PRP | Not included: no histomorphometric analysis |
| Simon et al., 2009 | PRF | Not included: animal study |
| Alissa et al., 2010 | PRP | Not included: no histomorphometric analysis |
| Celio-Mariano et al., 2012 | PRP | Not included: no histomorphometric analysis |
| Singh et al., 2012 | PRF | Not included: no histomorphometric analysis |
| Farina et al., 2013 | PRGF | Not included: short communication |
| Hauser et al., 2013 | PRF | Not included: follow-up less than 10 weeks |
| Rao et al., 2013 | PRF | Not included: no histomorphometric analysis |
| Suttapreyasri et al., 2013 | PRF | Not included: no histomorphometric analysis |
| Marenzi et al. 2015 | L-PRF | Not included: no histomorphometric analysis |
| Anwandter et al., 2016 | L-PRF | Not included: no histomorphometric analysis |
| Temmerman et al., 2016 | L-PRF | Not included: no histomorphometric analysis |
| Thakkar et al., 2016 | PRF | Not included: no histomorphometric analysis |
| Alzahrani et al., 2017 | PRF | Not included: no histomorphometric analysis |
| Clark et al., 2018 | A-PRF | Not included: incomplete data, mean and standard deviation are not reported in the study |
| El-Hamid et al., 2018 | PRGF | Not included: follow-up less than 10 weeks |
| Kumar et al., 2018 | PRF | Not included: no histomorphometric analysis |
| Srinivas et al., 2018 | PRF | Not included: no histomorphometric analysis |
| Zhang et al., 2018 | PRF | Not included: no randomization |
| Ahmed et al., 2019 | PRF | Not included: no histomorphometric analysis |
| Areewong et al., 2019 | PRF | Not included: follow-up less than 10 weeks |
| Arya et al., 2019 | PRGF | Not included: no histomorphometric analysis |
| Gnatek et al., 2019 | CGF | Not included: no histomorphometric analysis |
| Ritto et al., 2019 | L-PRF | Not included: no histomorphometric analysis |
| Badakhshan et al., 2020 | L-PRF | Not included: no histomorphometric analysis |
| Mozzati et al., 2020 | L-PRF | Not included: no histomorphometric analysis |
| Nisar et al., 2020 | PRP | Not included: no histomorphometric analysis |
| Sharma et al., 2020 | PRF | Not included: no histomorphometric analysis |
| Stumbras et al. 2020 (2) | PRGF | Not included: no histomorphometric analysis |
| Ustaoğlu et al., 2020 | L-PRF and T-PRF | Not included: no histomorphometric analysis |
| Aravena et al., 2021 | L-PRF | Not included: no histomorphometric analysis |
| Ma et al., 2021 | CGF | Not included: incomplete data, mean and standard deviation are not reported in the study |
| Alrayyes, 2022 | A-PRF | Not included: no histomorphometric analysis |
| Asoka, 2022 | PRF | Not included: no histomorphometric analysis |
| Brazdeikytė et al., 2022 | A-PRF and PRGF | Not included: no histomorphometric analysis |
| Fang, 2022 | CGF | Not included: no histomorphometric analysis |
| Ghanaati, 2022 | PRF | Not included: no histomorphometric analysis |
| Guadilla, 2022 | P-PRP | Not included: does not specify new bone percentage. |
| Ibrahim, 2022 | PRF | Not included: no histomorphometric analysis |
| Keramu, 2022 | CGF | Not included: no histomorphometric analysis |
| Liu, 2022 | CGF | Not included: no histomorphometric analysis |
| Mozzati, 2022 | CGF | Not included: no histomorphometric analysis |
| Niedzielska et al., 2022 | PRF | Not included: no histomorphometric analysis |
| Park, 2022 | L-PRF | Not included: animal study |
| Pereira, 2022 | A-PRF+ | Not included: no histomorphometric analysis |
| Rengarajoo, 2022 | Lyophilised PRF | Not included: no histomorphometric analysis |
| Saragayan, 2022 | PRP | Not included: no histomorphometric analysis |
| Shamshad, 2022 | PRF | Not included: no histomorphometric analysis |
| Shruti, 2022 | PRF | Not included: no histomorphometric analysis |
| Vicente Rodrigues, 2022 | PRF | Not included: no histomorphometric analysis |
| Wang, 2022 | L-PRF | Not included: no histomorphometric analysis |

**Supplemental Table 3:** Du Toit et al. CONSORT 2010 checklist of information to include when reporting a within-person randomised trial. For within-person trials, a group is the set of participants’ body sites that was allocated a particular intervention.

| **Section/Topic** | **Item no.** | **Standard CONSORT Checklist item** | **Extension for within-person trials** | **SCORE** |
| --- | --- | --- | --- | --- |
| **Title and abstract** | | | |  |
|  | 1a | Identification as a randomised trial in the title | Identification as a within-person randomised trial in the title | 2 |
|  | 1b | Structured summary of trial design, methods, results, and conclusions (for specific guidance see CONSORT for abstracts[3]) | Specify a within-person design and report all information outlined in table 2 | 0 |
| **Introduction** | | | |  |
| Background and objectives | 2a | Scientific background and explanation of rationale |  | 1 |
|  | 2b | Specific objectives or hypotheses |  | 1 |
| **Methods** | | | |  |
| Trial design | 3a | Description of trial design (such as parallel, factorial) including allocation ratio | Rationale for using a within-person design and identification of body sites | 1 |
|  | 3b | Important changes to methods after trial commencement (such as eligibility criteria), with reasons |  | N/A |
| Participants | 4a | Eligibility criteria for participants | Eligibility criteria for body sites | 2 |
|  | 4b | Settings and locations where the data were collected |  | 1 |
| Interventions | 5 | The interventions for each group with sufficient details to allow replication, including how and when they were actually administered | Whether interventions were given sequentially or concurrently | 2 |
| Outcomes | 6a | Completely defined pre-specified primary and secondary outcome measures, including how and when they were assessed | Outcomes should be clearly defined as per-site or per-person | 2 |
|  | 6b | Any changes to trial outcomes after the trial commenced, with reasons |  | N/A |
| Sample size | 7a | How sample size was determined | Report the correlation between body sites | 2 |
|  | 7b | When applicable, explanation of any interim analyses and stopping guidelines |  | N/A |
| Randomisation: | | | |  |
| Sequence generation | 8a | Method used to generate the random allocation sequence |  | 1 |
|  | 8b | Type of randomisation; details of any restriction (such as blocking and block size) | Methods used to determine the allocation sequence of body sites and treatments within an individual (e.g. how first site to be treated was decided) | 2 |
| Allocation concealment mechanism | 9 | Mechanism used to implement the random allocation sequence (such as sequentially numbered containers), describing any steps taken to conceal the sequence until interventions were assigned |  | 1 |
| Implement-ation | 10 | Who generated the random allocation sequence, who enrolled participants, and who assigned participants to interventions | Replaced by 10a | 1 |
|  | 10a |  | Who generated the random allocation sequence, who enrolled participants, and who assigned body sites to interventions | 1 |
| Blinding (masking) | 11a | If done, who was blinded after assignment to interventions (for example, participants, care providers, those assessing outcomes) and how |  | 1 |
|  | 11b | If relevant, description of the similarity of interventions |  | 1 |
| Statistical methods | 12a | Statistical methods used to compare groups for primary and secondary outcomes | Statistical methods appropriate for within-person design | 2 |
|  | 12b | Methods for additional analyses, such as subgroup analyses and adjusted analyses |  | 0 |
| **Results** | | | |  |
| Participant flow  (a diagram is strongly recommended) | 13a | For each group, the numbers of participants who were randomly assigned, received intended treatment, and were analysed for the primary outcome | Number of participants and number of body sites at each stage [See Figure 1] | 1 |
|  | 13b | For each group, losses and exclusions after randomisation, together with reasons | Number of participants and number of body sites lost or excluded at each stage, with reasons | 2 |
| Recruitment | 14a | Dates defining the periods of recruitment and follow-up |  | 0 |
|  | 14b | Why the trial ended or was stopped |  | N/A |
| Baseline data | 15 | A table showing baseline demographic and clinical characteristics for each group | Baseline characteristics for site and individual participants as applicable | 2 |
| Numbers analysed | 16 | For each group, number of participants (denominator) included in each analysis and whether the analysis was by original assigned groups | Number of randomised body sites in each group included in each analysis | 2 |
| Outcomes and estimation | 17a | For each primary and secondary outcome, results for each group, and the estimated effect size and its precision (such as 95% confidence interval) | Observed correlation between body sites for continuous outcomes and tabulation of paired results for binary outcomes | 2 |
|  | 17b | For binary outcomes, presentation of both absolute and relative effect sizes is recommended |  | N/A |
| Ancillary analyses | 18 | Results of any other analyses performed, including subgroup analyses and adjusted analyses, distinguishing pre-specified from exploratory |  | 2 |
| Harms | 19 | All important harms or unintended effects in each group (for specific guidance see CONSORT for harms) | Harms or unintended effects reported by participant and by body site | N/A |
| **Discussion** | | | |  |
| Limitations | 20 | Trial limitations, addressing sources of potential bias, imprecision, and, if relevant, multiplicity of analyses |  | 1 |
| Generalisability | 21 | Generalisability (external validity, applicability) of the trial findings |  | 1 |
| Interpretation | 22 | Interpretation consistent with results, balancing benefits and harms, and considering other relevant evidence |  | 1 |
| **Other information** | | | |  |
| Registration | 23 | Registration number and name of trial registry |  | 0 |
| Protocol | 24 | Where the full trial protocol can be accessed, if available |  | 0 |
| Funding | 25 | Sources of funding and other support (such as supply of drugs), role of funders |  | 0 |

From: Pandis N, Chung B, Scherer RW, Elbourne D, Altman DG. CONSORT 2010 statement: extension checklist for reporting within person randomised trials. BMJ. 2017;357.

*This document is from an Open Access article distributed in accordance with the Creative Commons Attribution Non Commercial (CC BY-NC 4.0) license, which permits others to distribute, remix, adapt, build upon this work non-commercially, and license their derivative works on different terms, provided the original work is properly cited and the use is non-commercial. See:*[*http://creativecommons.org/licenses/by-nc/4.0/*](http://creativecommons.org/licenses/by-nc/4.0/)*.*

**Supplemental Table 4:** Castro et al. CONSORT 2010 checklist of information to include when reporting a within-person randomised trial. For within-person trials, a group is the set of participants’ body sites that was allocated a particular intervention.

| **Section/Topic** | **Item no.** | **Standard CONSORT Checklist item** | **Extension for within-person trials** | **SCORE** |
| --- | --- | --- | --- | --- |
| **Title and abstract** | | | |  |
|  | 1a | Identification as a randomised trial in the title | Identification as a within-person randomised trial in the title | 2 |
|  | 1b | Structured summary of trial design, methods, results, and conclusions (for specific guidance see CONSORT for abstracts[3]) | Specify a within-person design and report all information outlined in table 2 | 0 |
| **Introduction** | | | |  |
| Background and objectives | 2a | Scientific background and explanation of rationale |  | 1 |
|  | 2b | Specific objectives or hypotheses |  | 1 |
| **Methods** | | | |  |
| Trial design | 3a | Description of trial design (such as parallel, factorial) including allocation ratio | Rationale for using a within-person design and identification of body sites | 0 |
|  | 3b | Important changes to methods after trial commencement (such as eligibility criteria), with reasons |  | N/A |
| Participants | 4a | Eligibility criteria for participants | Eligibility criteria for body sites | 2 |
|  | 4b | Settings and locations where the data were collected |  | 1 |
| Interventions | 5 | The interventions for each group with sufficient details to allow replication, including how and when they were actually administered | Whether interventions were given sequentially or concurrently | 2 |
| Outcomes | 6a | Completely defined pre-specified primary and secondary outcome measures, including how and when they were assessed | Outcomes should be clearly defined as per-site or per-person | 2 |
|  | 6b | Any changes to trial outcomes after the trial commenced, with reasons |  | N/A |
| Sample size | 7a | How sample size was determined | Report the correlation between body sites | 2 |
|  | 7b | When applicable, explanation of any interim analyses and stopping guidelines |  | N/A |
| Randomisation: | | | |  |
| Sequence generation | 8a | Method used to generate the random allocation sequence |  | 1 |
|  | 8b | Type of randomisation; details of any restriction (such as blocking and block size) | Methods used to determine the allocation sequence of body sites and treatments within an individual (e.g. how first site to be treated was decided) | 0 |
| Allocation concealment mechanism | 9 | Mechanism used to implement the random allocation sequence (such as sequentially numbered containers), describing any steps taken to conceal the sequence until interventions were assigned |  | 0 |
| Implement-ation | 10 | Who generated the random allocation sequence, who enrolled participants, and who assigned participants to interventions | Replaced by 10a | 0 |
|  | 10a |  | Who generated the random allocation sequence, who enrolled participants, and who assigned body sites to interventions | 0 |
| Blinding (masking) | 11a | If done, who was blinded after assignment to interventions (for example, participants, care providers, those assessing outcomes) and how |  | 1 |
|  | 11b | If relevant, description of the similarity of interventions |  | 0 |
| Statistical methods | 12a | Statistical methods used to compare groups for primary and secondary outcomes | Statistical methods appropriate for within-person design | 2 |
|  | 12b | Methods for additional analyses, such as subgroup analyses and adjusted analyses |  | 1 |
| **Results** | | | |  |
| Participant flow  (a diagram is strongly recommended) | 13a | For each group, the numbers of participants who were randomly assigned, received intended treatment, and were analysed for the primary outcome | Number of participants and number of body sites at each stage [See Figure 1] | 2 |
|  | 13b | For each group, losses and exclusions after randomisation, together with reasons | Number of participants and number of body sites lost or excluded at each stage, with reasons | 2 |
| Recruitment | 14a | Dates defining the periods of recruitment and follow-up |  | 1 |
|  | 14b | Why the trial ended or was stopped |  | N/A |
| Baseline data | 15 | A table showing baseline demographic and clinical characteristics for each group | Baseline characteristics for site and individual participants as applicable | 2 |
| Numbers analysed | 16 | For each group, number of participants (denominator) included in each analysis and whether the analysis was by original assigned groups | Number of randomised body sites in each group included in each analysis | 2 |
| Outcomes and estimation | 17a | For each primary and secondary outcome, results for each group, and the estimated effect size and its precision (such as 95% confidence interval) | Observed correlation between body sites for continuous outcomes and tabulation of paired results for binary outcomes | 2 |
|  | 17b | For binary outcomes, presentation of both absolute and relative effect sizes is recommended |  | N/A |
| Ancillary analyses | 18 | Results of any other analyses performed, including subgroup analyses and adjusted analyses, distinguishing pre-specified from exploratory |  | 1 |
| Harms | 19 | All important harms or unintended effects in each group (for specific guidance see CONSORT for harms) | Harms or unintended effects reported by participant and by body site | N/A |
| **Discussion** | | | |  |
| Limitations | 20 | Trial limitations, addressing sources of potential bias, imprecision, and, if relevant, multiplicity of analyses |  | 1 |
| Generalisability | 21 | Generalisability (external validity, applicability) of the trial findings |  | 1 |
| Interpretation | 22 | Interpretation consistent with results, balancing benefits and harms, and considering other relevant evidence |  | 1 |
| **Other information** | | | |  |
| Registration | 23 | Registration number and name of trial registry |  | 1 |
| Protocol | 24 | Where the full trial protocol can be accessed, if available |  | 1 |
| Funding | 25 | Sources of funding and other support (such as supply of drugs), role of funders |  | 1 |

From: Pandis N, Chung B, Scherer RW, Elbourne D, Altman DG. CONSORT 2010 statement: extension checklist for reporting within person randomised trials. BMJ. 2017;357.

*This document is from an Open Access article distributed in accordance with the Creative Commons Attribution Non Commercial (CC BY-NC 4.0) license, which permits others to distribute, remix, adapt, build upon this work non-commercially, and license their derivative works on different terms, provided the original work is properly cited and the use is non-commercial. See:*[*http://creativecommons.org/licenses/by-nc/4.0/*](http://creativecommons.org/licenses/by-nc/4.0/)*.*

**Supplemental Figure 1:** Forest plots depicting overall standardized mean difference estimation for meta-analysis comparing PCs group vs spontaneous healing (subgroup analysis for study design RCT vs RCT-split mouth).


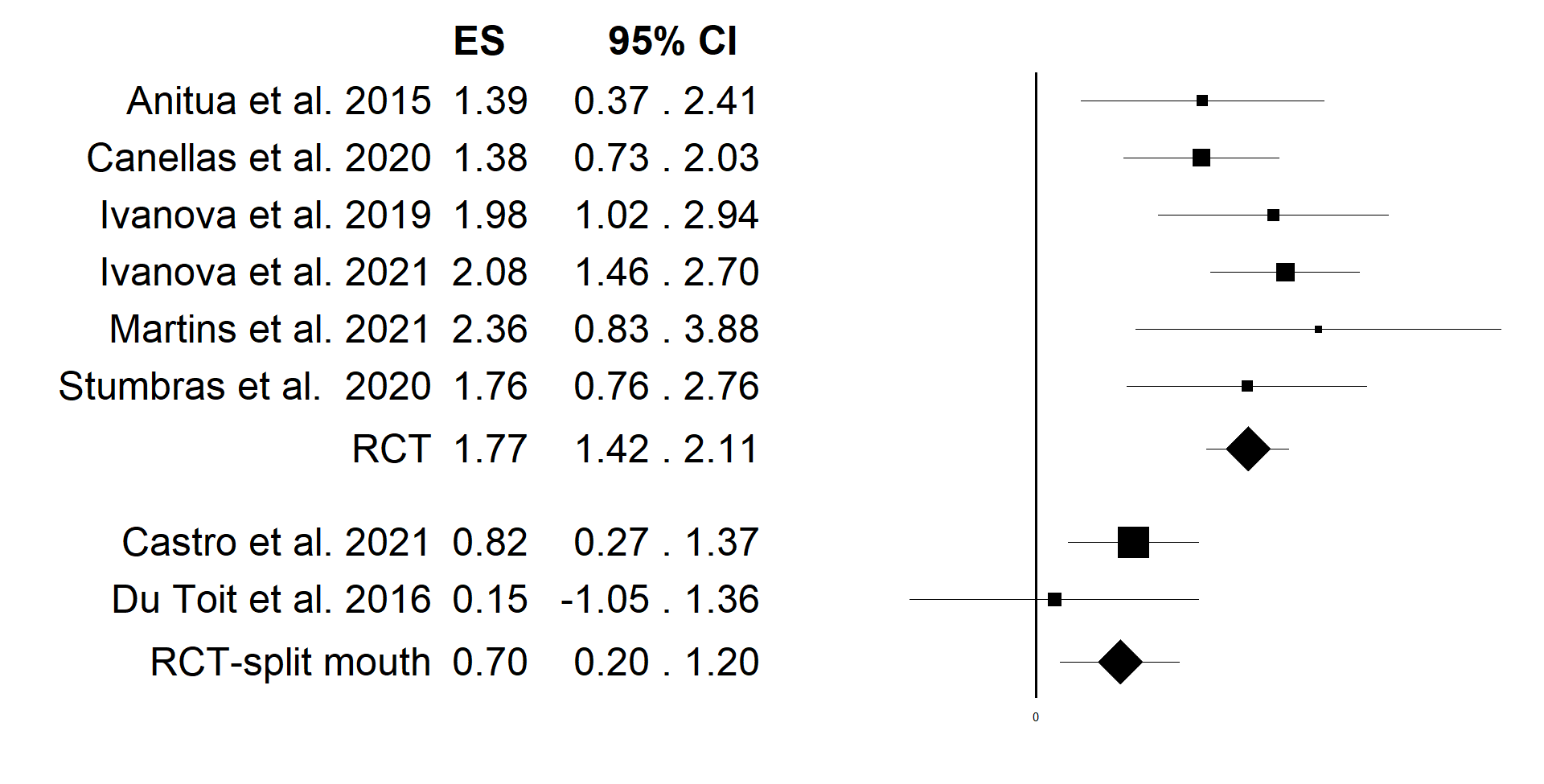


**Supplemental Figure 2:** Forest plots depicting overall standardized mean difference estimation for meta-analysis comparing PCs group vs spontaneous healing (subgroup analysis for L-PRF vs P-PRP).


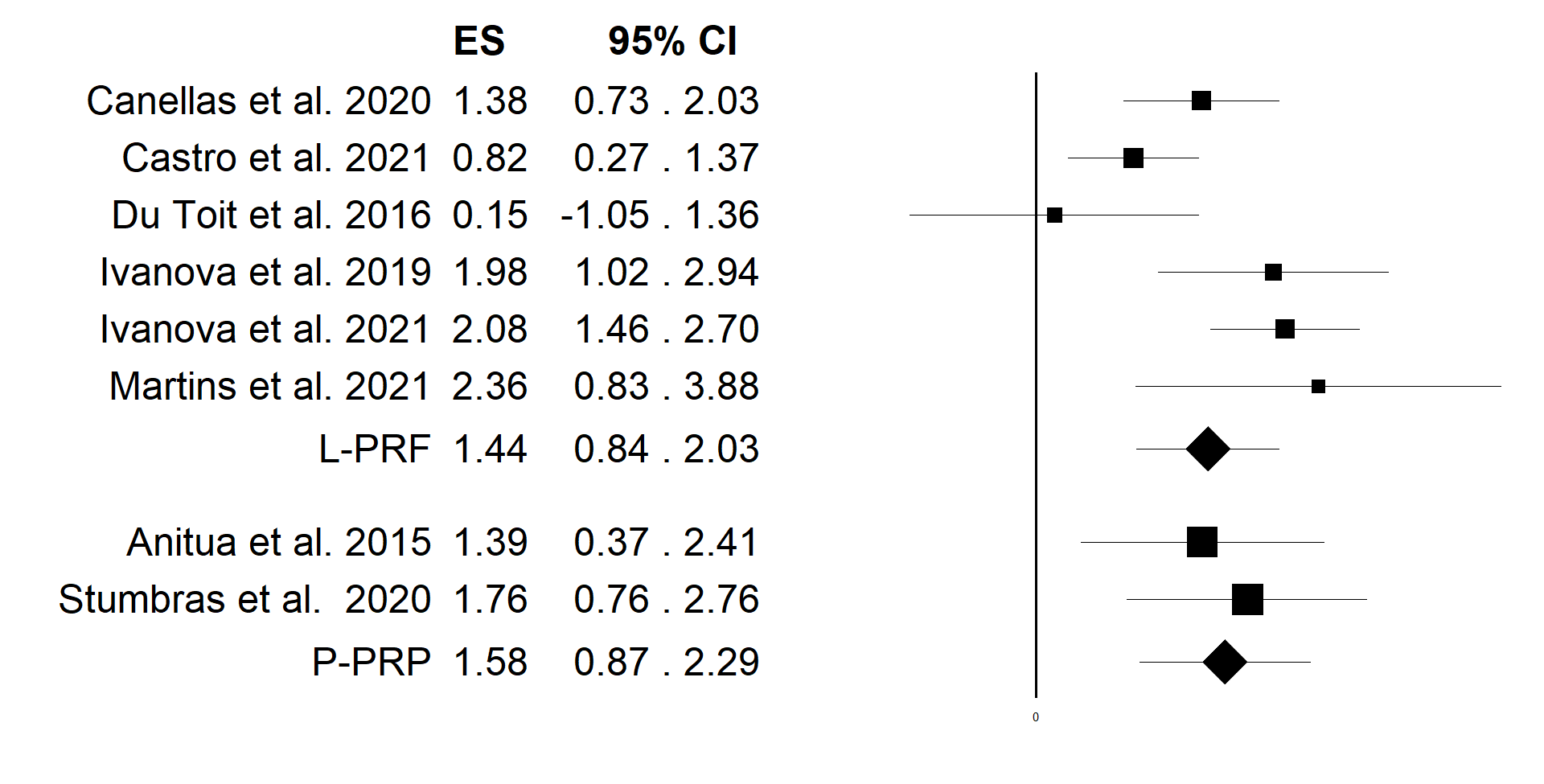

Supplement: Supplementary file 1 — Supplementary file1 (DOCX 133 KB) [file 784_2023_5126_MOESM1_ESM.docx]
